# Predictors of glycemic control among type 1 pediatric diabetes patients—Northeast Ethiopia

**DOI:** 10.3389/fcdhc.2024.1449641

**Published:** 2025-01-17

**Authors:** Hiwot Adege, Gedefaw Getnet, Abyou Seyfu Ambaye, Mekuanint Terefe Kassa, Bedilu Linger Endalifer

**Affiliations:** ^1^ School of Medicine, College of Medicine and Health Science, Wollo University, Dessie, Ethiopia; ^2^ School of Pharmacy, College of Medicine and Health Science, Wollo University, Dessie, Ethiopia; ^3^ Department of Pharmacy, Asrat Woldeyes Health Science Campus, Debre Berhan University, Debre Berhan, Ethiopia; ^4^ School of Medicine, Asrat Woldeyes Health Science Campus, Debre Berhan University, Debre Berhan, Ethiopia

**Keywords:** glycemic control, pediatrics, type 1 DM, northeast, Ethiopia

## Abstract

**Introduction:**

Type 1 diabetes mellitus is the most common endocrine–metabolic disorder in children and adolescents worldwide. This study aims to determine the metabolic glycemic control levels and predictors among pediatric type 1 diabetes patients.

**Methods:**

An institution-based prospective cross-sectional study was conducted from July 01, 2022 to October 30, 2022 among patients attending pediatric chronic follow-up at Dessie Comprehensive Specialized Hospital, Northeast Ethiopia. Patients on follow-up for more than 3 months and aged under 18 years were enrolled in the study consecutively based on their visits. Data were entered into Epidata version 3.1 and exported to SPSS version 26 for analysis. Bivariate and multivariate analyses were done to identify the factors affecting glycemic control.

**Result:**

Among 203 patients with type 1 diabetes, the average age was 8.72 ± 4.25 years, with the largest group of participants being over 10 years old (77 patients, 37%). The majority of the patients were female, totaling 126 (62.1%), and about 69 (34.0%) were in grades 7–10. Retinopathy, nephropathy, and diabetic ketoacidosis were commonly observed diabetic-related complications in this study. More than half of the patients, 107 (52.7%), had good diabetic control, while 96 (47.3%) had poor diabetic control. Factors significantly associated with poor glycemic control include having diabetes for more than 5 years (aOR: 1.45; 95% CI: 0.47, 0.91), being a third-born child (aOR: 0.22; 95% CI: 0.05, 0.92), having a comorbid disease condition (aOR: 1.84; 95% CI: 0.29, 0.92), and experiencing diabetes-related complications (aOR: 0.26; 95% CI: 0.08, 0.97).

**Conclusion:**

The study found that glycemic control is significantly poor among pediatric patients with type 1 diabetes. As a result, these patients require special attention to prevent the long-term consequences of diabetes.

## Introduction

Diabetes is a metabolic condition characterized by chronic hyperglycemia, resulting from either an absolute or a relative deficiency of insulin, impaired insulin action, or both (1, 2). Type 1 diabetes mellitus (T1DM) is a prevalent endocrine–metabolic disorder among children and adolescents globally. In the USA, the prevalence is 190 per 100,000 school-aged children, with annual incidence rates varying from 1.7 per 100,000 in China (3) to 40 per 100,000 in Finland (4). Over the past 30 years, the incidence of T1DM has been increasing globally (3–5); in some African countries, the incidence ranged from 4.4 per 100,000 in Algeria to 20 per 100,000 in Morocco (6). Whereas in sub-Saharan Africa few studies have been conducted, estimates from Sudan indicate an increase in incidence from 9.5 per 100,000 in 1991 to 10.3 per 100,000 in 1999 (7). A study conducted in Ethiopia found that the average HbA1c level among children was 9.7%, with the majority (85.2%) of the children exhibiting poor glycemic control, defined as a serum HbA1c level greater than 7.5% (8).

Glycemic control is the ultimate goal of diabetes management (9, 10). Type 1 diabetes mellitus is associated with serious macrovascular and microvascular complications, such as nephropathy, retinopathy, and neuropathy (11). Due to the risk of hypoglycemia unawareness in children, the American Diabetes Association (ADA) recommends an HbA1c range of 7.5% to 8.5% for children under 6 years old. For children aged 6 to 12 years, an HbA1c level of less than 8% is advised, while for those over 12 years, a level below 7.5% is considered optimal (12). Various target levels for HbA1c have been proposed, with the goal being to achieve the lowest possible HbA1c without raising the risk of hypoglycemia. Currently, the International Society for Pediatric and Adolescent Diabetes (ISPAD) recommends a target HbA1c of less than 7.5% (58 mmol/mol) for all age groups (13).

Different factors may contribute to poor metabolic glycemic control, such as unmet basic food needs, mothers’ low level of education (14), being female, having a greater body mass index, and low medication adherence (15). Additionally, a duration of diabetes >8 years, diabetes complications, and poor self-care behavior were significant predictors of poor glycemic control (16). Factors contributing to poor glycemic control in children with T1DM include insulin dose determination and administration as well as factors such as being separated from their mothers, being over 1 year old, having diabetes for more than 5 years, and having elevated serum triglyceride levels (1).

To prevent complications, it is recommended to begin screening for albuminuria through urine analysis to detect diabetic nephropathy, conduct annual eye examinations for diabetic retinopathy, and screen for peripheral neuropathy starting at the age of 11 for those with 2–5 years of diabetes duration. These screenings should be repeated annually thereafter (17). Despite different strategies being applied to prevent complications, good glycemic control is a crucial parameter (18). This study focused on the level of glycemic control and the factors that predict glycemic control among patients with T1DM.

## Methods

### Study area and study design

The study was conducted at the South Wollo Dessie Comprehensive Specialized Hospital, located in Dessie, a city in northeastern Ethiopia, 401 km north of Addis Ababa and 476 km east of Bahir Dar. The hospital serves over 10 million people from the South Wollo zone and other regions of Ethiopia. It operates a follow-up clinic three times a week—on Mondays, Wednesdays, and Fridays. A facility-based cross-sectional study was conducted involving all pediatric diabetic patients attending follow-up appointments from July 1, 2022 to October 30, 2022.

### Sample size determination and sampling technique

During the study period, 203 pediatric patients with type 1 diabetes met the selection criteria. The study was conducted over four 4-month periods, with the patients returning for follow-up visits every 1 to 3 months. All patients who attended follow-up visits during the study period were included in the study, with the participants selected based on their convenient visit times.

### Eligibility criteria

Children and adolescents with diabetes who have been on insulin treatment for ≥3 months of age and age <18 years were included in the study.

### Variables of the study

Metabolic–glycemic control, either good or poor control level, is the dependent variable.

### Independent variables

Demographic factors: age, sex, address, level of education of the caregiver, occupation of the caregiver, marital status of the caregiver, family history of diabetes, and socioeconomic state of the family.

Disease-related characteristics: age at onset of disease, duration of the disease, type and frequency of insulin injection, presence of comorbidity, diabetic complication, types of insulin, doses of insulin, injection site, and frequency of checkup are independent variables.

### Data collection tool and procedures

The patients and caregivers were interviewed, and the patients’ medical records were reviewed to gather relevant clinical information from their charts. A data collection tool was developed to include questions about sociodemographic details and diabetes-related information. Sociodemographic data collected included age, sex, address, primary caregiver, level of parental/caregiver education, and the occupation of the parents/caregivers.

Data on diabetes-related information were collected, including age at diagnosis, condition at initial presentation, duration of illness, insulin regimen, frequency of insulin injections, total daily insulin dose, and number of meals per day. The presence of diabetes-related complications was assessed through the patients’ medical history, physical exams, and relevant investigations. In the diabetes clinic, children were screened for chronic complications according to the screening criteria set by the American Diabetes Association (ADA) (19). Diabetic retinopathy was evaluated using fundus photography to assess for hemorrhages, edema, new vessel formation, and exudates in the eye and was performed by an ophthalmologist at the ophthalmology clinic, which operates on Mondays through Fridays. Diabetic neuropathy was assessed by screening for symptoms and signs of neuropathy, along with a comprehensive foot examination. HbA1c tests were conducted at the DCSH laboratory unit during the patients’ routine follow-up visits. A less stringent HbA1c goal of <7.5% was used to classify patients as having good or poor glycemic control. Specifically, an HbA1c value <7.5% was considered indicative of good glycemic control, while a value ≥7.5% (58 mmol/mol) was regarded as indicative of poor control (13).

### Data quality control

The questionnaire was initially developed in English, then translated into Amharic, and subsequently back-translated into English to ensure consistency and accuracy. The language translation was carried out by two research experts familiar with the study area and who were proficient in both the local language and culture. They independently translated the text from English to Amharic and *vice versa*. A pretest was conducted with 5% of the total sample size at Borumeda Hospital, and necessary changes and modifications were made to the questionnaire based on the feedback from the pretest. Data collectors received 3 days of training on the entire data collection process. During the data collection period, supervisors visited the study sites on each follow-up day to review the completed questionnaires for completeness, accuracy, and clarity.

### Data processing and analysis

Data were entered using EpiData version 3.1 for cleaning and then exported to SPSS version 26 for analysis. Descriptive statistics were performed for all variables. Bivariable and multivariable binary logistic regression analyses were used to identify factors associated with glycemic control. Bivariate regression was employed to examine the association between dependent and independent variables. During the bivariate analysis, variables with *p*-values less than 0.25 were included in the multivariable analysis. The direction and strength of the associations were determined using crude odds ratios and adjusted odds ratios, along with their 95% CIs. In the multivariable analysis, variables with *p*-values less than 0.05 were considered statistically significant for the outcome variable. Hosmer and Lemeshow’s test was used to assess whether the independent variables adequately predicted the outcome variable. Variables were considered statistically significant if the *p*-value was less than 0.05. In addition to identifying significant variables, interactions and potential confounders were examined during the model-building process.

## Result

A total of 203 patients with type 1 diabetes had an average age of 8.72 ± 4.25 years, with the highest proportion (77, 37%) of participants being above 10 years of age. The majority of the patients were female, with 126 (62.1%) being girls. Approximately 69 (34.0%) of the patients were in grades 7 to 10. About 82 (40.4%) were first-born children, and 186 (91.6%) lived with their parents. Of those, 138 (68.0%) had their mothers as the primary caregivers (**Table 1**).

**Table 1:** Sociodemographic characteristics of the study participants in Northeast Ethiopia (*N* = 203).

Variable	Category	Frequency	Percentage
Age	≤5	65	32.0
	5–10	61	30.0
	>10	77	37.9
	Mean ± SD: 8.7215 ± 4.25061		
Sex	Male	77	37.9
	Female	126	62.1
Residence	Rural	113	55.7
	Urban	90	44.3
Education level of the patient	KG not started	41	20.2
	KG	35	17.2
	Grades 1–6	58	28.6
	Grades 7–10	69	34.0
Birth order of the baby	1st baby	82	40.4
	2nd baby	52	25.6
	3rd baby	24	11.8
	4th and above	45	22.2
Family structure	Parent family	186	91.6
	Single parent	9	4.4
	Not living with the parent	8	3.9
Primary caregiver	Mother	138	68.0
	Father	30	14.8
	Sibling	16	7.9
	Other	19	9.4
Education level of care giver	No formal	97	47.8
	Elementary	61	30.0
	High school	30	14.8
	College/university	15	7.4
Occupation of the caregiver	Daily laborer	35	17.2
	Merchant	82	40.4
	Farmer	59	29.1
	Employee	27	13.3
Family history of diabetes	Yes	29	14.3
	No	174	85.7
Adherence to diabetic care	No	182	89.7
	Yes	21	10.3

KG, kindergarten.

### Drug and disease characteristics

Most patients (94, 46.3%) were diagnosed with diabetes at ages 5 to 10, while the duration of diabetes for 109 (53.7%) patients ranged from 2 to 5 years. Regarding the initial presentation, around 129 (63.5%) patients presented with various diabetic symptoms at the time of diagnosis. A total of 107 (52.7%) patients achieved adequate metabolic control (HbA1c <7.5%). Among all participants, 142 (70.0%) had regular follow-up visits. Additionally, diabetic-related complications were observed in the participants, with seven (3.4%) having retinopathy, nine (4.4%) with nephropathy, and 10 (4.9%) experiencing diabetic ketoacidosis (**Table 2**).

**Table 2:** Disease and drug-related characteristics of the study participants in Northeast Ethiopia (*N* = 203).

Variable	Category	Frequency	Percentage
Age at diagnosis	<5	82	40.4
	5–10	94	46.3
	>10	27	13.3
Duration of diabetes	<2 years	34	16.7
	2–5 years	109	53.7
	>5 years	60	29.6
Initial presentation	DM symptoms	129	63.5
	DKA	72	35.5
	Incidental	2	1.0
Insulin formulation	Mixed (lente+ regular)	191	94.1
	NPH + RI	4	2.0
	NPH only	8	3.9
Diabetes-related complications	No	177	87.2
	Yes	26	12.8
Types of complications	No	177	87.2
	Retinopathy	7	3.4
	Nephropathy	9	4.4
	DKA	10	4.9
Comorbidity	Yes	68	33.5
	No	135	66.5
Comorbidities	HTN	18	8.9
	Cardiac	3	1.5
	Renal	47	23.2
	No	135	66.5
Daily insulin dose	Less than 15 IU	123	60.6
	15–30 IU	67	33.0
	Greater than 30 IU	13	6.4
Frequency of self-monitoring per month	No	143	70.4
	Once	37	18.2
	Twice	23	11.3
Family structure	Parent family	186	91.6
	Single parent	9	4.4
	Not living with the parent	8	3.9
Injection site complication	Yes	29	14.3
	No	174	85.7
Parents can afford	Yes	105	51.7
	No	98	48.3
Average number of meals	≤3	92	45.3
	≥4	111	54.7
Use of refined sugar	Yes	22	10.8
	No	181	89.2
Insulin injector	Patient	91	44.8
	Parent	112	55.2
Missed insulin	Yes	13	6.4
	No	190	93.6
Hgb A1C category	<7.5	107	52.7
	7.49–9.5	68	33.5
	>9.5	28	13.8
Number of hospitalization category	No	107	52.7
	≤2	57	28.1
	Three and above	39	19.2
Medical follow-up	Regular	142	70.0
	Irregular	61	30.0

RI, regular insulin; NPH, isophane insulin; IU, international unit.

### Glycemic control

**Figure 1:**
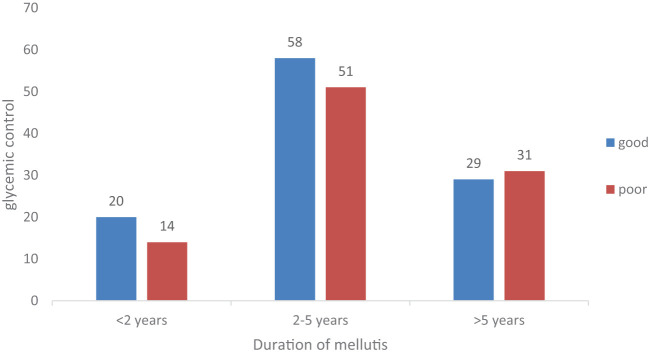
Distribution of glycemic control with the age of the patients (*N* = 203).

Among the participants, 107 (52.7%) had good diabetic control, while 96 (47.3%) had poor glycemic control. Poor glycemic control was more prevalent in patients with 2–5 years of diabetes duration, accounting for 52 (25.1%) of the participants (**Figure 1**).

### Predictors of glycemic control

The logistic regression analysis of factors associated with glycemic control revealed that the education level of patients, birth order, education level of the caregiver, duration of diabetes, diabetes-related complications, comorbidities, and daily insulin dose were variables significantly associated with poor glycemic control, with *p*-values <0.25 (**Table 3**). All variables that showed significant associations in the bivariate logistic analysis were further analyzed using multivariable logistic regression. In the multivariable analysis, three variables were found to be significantly associated with poor glycemic control: being a third-born child, having diabetes-related complications, and having other comorbid conditions.

**Table 3:** Predictors of poor glycemic control in the study participants—Northeast Ethiopia (*N* = 203).

Variable		Diabetes control		COR (95% CI)	aOR (95% CI)
		Good (N, P)	Poor (N, P)		
Education level of the patients	KG not started	19 (9.4)	22 (10.8)	1.16 (0.81, 1.66)	1.71 (0.63, 4.62)
	KG	24 (11.8)	11 (5.4)	0.46 (0.30, 0.70)	0.52 (0.18, 1.50)
	Grades 1–6	31 (15.3)	27 (13.3)	0.87 (0.64, 1.18)	0.55 (0.25, 1.22)
	Grades 7–10	33 (16.3)	36 (17.7)	1	1
Birth order of the baby	1st	40 (19.7)	42 (20.7)	1	1
	2nd	25 (12.3)	27 13.3)	1.08 (0.79, 1.49)	0.81 (0.35, 1.84)
	3rd^a^	16 (7.9)	8 (3.9)	0.50 (0.30, 0.82)	0.22 (0.05, 0.92)
	4th and above	26 (12.8)	19 (9.4)	0.73 (0.516, 1.03)	0.58 (0.25, 1.33)
Education level of the caregiver	No formal	54 (26.6)	43 (21.2)	1	1
	Elementary	26 (12.8)	35 (17.2)	1.35 (0.99, 1.81)	2.76 (0.93, 8.22)
	High school	16 (7.9)	14 (6.9)	0.88 (0.57, 1.33)	1.82 (0.58, 5.73)
	College/university	11 (5.4)	4 (2)	0.36 (0.19, 0.71)	1.14 (0.40, 3.23)
Duration of diabetes	<2 years	20 (9.8)	14 (6.9)	1	1
	2–5 years	58 (28.6)	51 (25.1)	0.70 (0.35, 1.39)	1.14 (0.40, 3.23)
	>5 years^a^	29 (14.3)	31 (15.3)	0.88 (0.30, 0.98)	1.45 (0.47, 0.91)
Diabetes-related complications	Yes^a^	18 (8.9)	8 (3.9)	0.40 (0.08, 0.96)	0.285 (0.08, 0.97)
	No	89 (43.8)	88 (43.4)	1	1
Comorbidities	Yes^a^	36 (17.7)	32 (15.8)	0.39 (0.14, 1.08)	1.84 (0.29, 0.92)
	No	71 (35)	64 (31.5)	1	1
Daily insulin dose	Less than 15 IU	74 (36.5)	51 (25.1)	1	1
	15–30 IU	25 (12.3)	40 (19.7)	0.69 (0.56, 0.85)	1.77 (0.78, 4.02)
	Greater than 30 IU	8 (3.9)	5 (2.5)	1.60 (1.19, 2.15)	0.74 (0.19, 2.83)

KG, kindergarten.

a Significant association.

Patients with diabetes for more than 5 years were 1.45 times more likely to experience poor glycemic control compared to those with less than 2 years of diabetes duration. Similarly, the presence of comorbid conditions increased the likelihood of poor glycemic control by 1.84 times. Being the third-born child was associated with 78% higher probability of poor glycemic control compared to being the first-born. Conversely, patients who developed diabetes-related complications had 71.5% lower probability of poor glycemic control (**Table 3**).

## Discussion

The results of this study suggest that in pediatric patients with T1DM, the level of glucose control was determined based on a glycosylated hemoglobin (HbA1c) level of ≥7.5% as indicative of poor control, and efforts were made to identify the factors contributing to poor control. This study assessed the extent of poor glycemic control and identified the factors contributing to it among children with diabetes attending routine follow-ups in northeastern Ethiopia.

According to this study, out of the total sample of 203 participants, 107 (52.7%) had good glycemic control, while 96 (47.3%) had poor glycemic control. This finding was compared with a previous study conducted in Egypt, which reported that 45.8% of the participants had poor glycemic control (20). This figure is lower than the previous study report in Hawasa in southern Ethiopia (83.6%) (18), in Harar in eastern Ethiopia (71.9%) (21), in Addis Ababa, Ethiopia (85.2%) (8), in Sudan (76%) (22), and in Iran (23). However, this figure is slightly higher compared to a finding in northwest Ethiopia (39.3%) (24). The discrepancy in the proportion of patients with poor glycemic control may be attributed to differences in the study setting, study design, sample size, and the level of diabetic care provided. Additionally, factors such as variations in the population served by the study setting, the education level of caregivers, and the availability of resources like insulin may also contribute to this difference.

Focusing on the determinants of poor glycemic control, the birth order of the child, specifically being a third-born, was found to have a significant role in poor glucose control (aOR: 0.22; 95% CI: 0.05, 0.92). Interestingly, being a third-born child appeared to have a protective effect against poor glycemic control. This could be explained by parental caregiving behavior, as parents may become more experienced and attentive in managing diabetes with subsequent children. The increased caregiving practices and attention toward the third child might result in better glycemic control compared to the firstborn.

Patients with diabetes for more than 5 years had an increased probability of poor glycemic control (aOR: 1.45; 95% CI: 0.47, 0.91). This finding is consistent with previous studies, which have suggested that a longer duration of diabetes is associated with a higher likelihood of poor glycemic control in pediatric patients. As the disease progresses, managing blood glucose levels effectively can become more challenging, potentially leading to poorer control over time (8, 25). In addition, the increased duration of diabetes may lead to financial challenges for families, as parents may struggle to afford the cost of medications (such as insulin) and ongoing care. This is supported by the findings of Shibeshi et al., which indicated that families who cannot afford insulin were more likely to have poor glycemic control. Financial constraints can limit access to necessary treatments and regular follow-up care, contributing to suboptimal diabetes management (18). Furthermore, the patients’ and caregivers’ attention on the disease condition may decrease, and they may consider it as a normal condition.

Indeed the presence of comorbid disease conditions (aOR: 1.84; 95% CI: 0.29, 0.92) was significantly associated with poor glycemic control, while diabetes-related complications (AOR: 0.26; 95% CI: 0.08, 0.97) were linked to better glycemic control. This may be explained by the increased attention and care that parents, caregivers, and patients themselves tend to provide once diabetes-related complications such as diabetic ketoacidosis (DKA), retinopathy, and nephropathy develop. The recognition of these serious complications often prompts more diligent management of the disease, resulting in improved glycemic control.

The strengths of the study include its observational design, the involvement of trained data collectors, and a relatively long data collection period. However, the study’s limitations include its single-center, cross-sectional nature, which restricts the generalizability of the findings. Therefore, the researchers suggest that future studies adopt a more robust cohort and follow-up design as well as a multicenter approach. Additionally, exploring other continuous glucose monitoring metrics would help develop stronger scientific evidence.

## Conclusion

In conclusion, glycemic control is significantly poor among the pediatric patients with T1DM in this study. The findings indicate that a longer duration of diabetes mellitus and the presence of comorbid disease conditions are associated with poorer glycemic control. Therefore, special attention and focused care are essential for these patients to prevent diabetes-related consequences and complications.

## Data Availability

The raw data supporting the conclusions of this article will be made available by the authors, without undue reservation.

## References

[1] WHO. Diagnosis of diabetes mellitus and intermediate hyperglycemia: report of a WHO/IDF consultation Vol. 3. Geneva: World Health Organization (2006).

[2] Saeedi P Petersohn I Salpea P Malanda B Karuranga S Unwin N. Global and regional diabetes prevalence estimates for 2019 and projections for 2030 and 2045: Results from the International Diabetes Federation Diabetes Atlas. Diabetes Res. Clin. pract. (2019) 157:107843. doi: 10.1016/j.diabres.2019.107843 31518657

[3] Gong C Meng X Jiang Y Wang X Cui H Chen X. Trends in childhood type 1 diabetes mellitus incidence in Beijing from 1995 to 2010: a retrospective multicenter study based on hospitalization data. Diabetes Technol. Ther. (2015) 17:159–65. doi: 10.1089/dia.2014.0205 25545069

[4] Tuomilehto J Virtala E Karvonen M Lounamaa R Pitkāniemi J Reunanen A. Increase in incidence of insulin-dependent diabetes mellitus among children in Finland. Int. J. Epidemiol. (1995) 24:984–92. doi: 10.1093/ije/24.5.984 8557457

[5] Dabelea D Bell RA D’Agostino RB Jr Imperatore G Johansen JM Linder B. Incidence of diabetes in youth in the United States. Jama. (2007) 297:2716–24. doi: 10.1001/jama.297.24.2716 17595272

[6] Ngwiri T Were F Predieri B Ngugi P Iughetti L. Glycemic control in Kenyan children and adolescents with type 1 diabetes mellitus. Int. J. Endocrinol. (2015) 2015:1–7. doi: 10.1155/2015/761759 PMC4606130 26494998

[7] Swai A Lutale JL McLarty DG. Prospective study of incidence of juvenile diabetes mellitus over 10 years in Dar es Salaam, Tanzania. Br. Med. J. (1993) 306:1570–2. doi: 10.1136/bmj.306.6892.1570 PMC1678021 8329915

[8] Fantahun B Leulseged TW. Glycemic control among children with type 1 diabetes mellitus and its determinants in a resource-limited setting. J. Pediatr. Endocrinol. Metab. (2022) 35:813–7. doi: 10.1515/jpem-2022-0144 35538692

[9] ADA. Classification and diagnosis of diabetes: standards of medical care in diabetes—2019. Diabetes Care. (2019) 42:S13–28. doi: 10.2337/dc19-S002 30559228

[10] Imran SA Agarwal G HS B Ross S. Targets for glycemic control. Can. J. diabet. (2018) 42:S42–S6. doi: 10.1016/j.jcjd.2017.10.030 29650110

[11] Brink SJ. Complications of pediatric and adolescent type 1 diabetes mellitus. Curr. Diabetes Rep. (2001) 1:47–55. doi: 10.1007/s11892-001-0010-1 12762957

[12] ADA. Children and Adolescents: standard of care in diabetes. Diabetes Care. (2023) 46:S230–S253. doi: 10.2337/dc23-Sint 36507640 PMC9810473

[13] Rewers M Pihoker C Donaghue K Hanas R Swift P Klingensmith GJ. Assessment and monitoring of glycemic control in children and adolescents with diabetes. Pediatr. diabet. (2009) 10:71–81. doi: 10.1111/j.1399-5448.2009.00582.x 19754620

[14] Araujo MB Mazza CS. Assessment of risk factors of poor metabolic control in type 1 diabetic children assisted in a public hospital in Argentina. Pediatr. diabet. (2008) 9:480–7. doi: 10.1111/j.1399-5448.2008.00388.x 18761645

[15] Demoz GT Gebremariam A Yifter H Alebachew M Niriayo YL Gebreslassie G. Predictors of poor glycemic control among patients with type 2 diabetes on follow-up care at a tertiary healthcare setting in Ethiopia. BMC Res. notes. (2019) 12:1–7. doi: 10.1186/s13104-019-4248-6 30947749 PMC6449968

[16] Oluma A Abadiga M Mosisa G Etafa W. Magnitude and predictors of poor glycemic control among patients with diabetes attending public hospitals of Western Ethiopia. PloS One. (2021) 16:e0247634. doi: 10.1371/journal.pone.0247634 33630936 PMC7906479

[17] Codner E Acerini CL Craig ME Hofer SE Maahs DM. ISPAD Clinical Practice Consensus Guidelines 2018: limited care guidance appendix. Pediatr. Diabetes. (2018) 19:328–38. doi: 10.1111/pedi.12767 30276975

[18] Shibeshi MS Daba AK Meiso KM Tadesse BT. Glycemic control among children and adolescents with diabetes in Southern Ethiopia: a cross-sectional study. BMC Endocr Disord. (2022) 22:161. doi: 10.1186/s12902-022-01070-y 35705956 PMC9202171

[19] ADA. Children and adolescents: standards of medical care in diabetes– 2020. Diabetes Care. (2020) 43:S163–S82. doi: 10.2337/dc20-S013 31862756

[20] Mohammad HA Farghaly HS Metwalley KA Monazea EM Abd El-Hafeez HA. Predictors of glycemic control in children with Type 1 diabetes mellitus in Assiut-Egypt. Indian J. Endocrinol. Metab. (2012) 16:796–802. doi: 10.4103/2230-8210.100679 23087867 PMC3475907

[21] Habteyohans BD Hailu BS Meseret F Mohammed A Berhanu Y Alemu A. Poor glycemic control and its associated factors among children with type 1 diabetes mellitus in Harar, eastern Ethiopia: A cross-sectional study. BMC endocr Disord. (2023) 23:208. doi: 10.1186/s12902-023-01453-9 37759193 PMC10538014

[22] Taha Z Eltoum Z Washi S. Predictors of glucose control in children and adolescents with type 1 diabetes: results of a cross-sectional study in Khartoum, Sudan. Open Access Macedonian J. Med. Sci. (2018) 6:2035–9. doi: 10.3889/oamjms.2018.423 PMC6290430 30559856

[23] Ghazaiean M Najafi B Zamanfar D Alipour MJ. Risk factors for suboptimal glycemic control in pediatrics with type 1 diabetes mellitus: a cross-sectional study. Sci. Rep. (2024) 14:7492. doi: 10.1038/s41598-024-57205-9 38553464 PMC10980686

[24] Kidie AA Ayal BG Ayele T Fentie EA Lakew AM. Poor glycemic control and associated factors among pediatric diabetes mellitus patients in northwest Ethiopia, 2020: facility-based cross sectional retrospective study design. Sci. Rep. (2022) 12:15664. doi: 10.1038/s41598-022-19909-8 36123389 PMC9485249

[25] Alassaf A Odeh R Gharaibeh L Ibrahim S Ajlouni K. Personal and clinical predictors of poor metabolic control in children with type 1 diabetes in Jordan. J. Diabetes Res. (2019) 2019:4039792. doi: 10.1155/2019/4039792 31355293 PMC6637667

